# Antibodies Biotinylated Using a Synthetic Z-domain from Protein A Provide Stringent In Situ Protein Detection

**DOI:** 10.1369/0022155413502360

**Published:** 2013-11

**Authors:** Sandra Andersson, Anna Konrad, Nikhil Ashok, Fredrik Pontén, Sophia Hober, Anna Asplund

**Affiliations:** Department of Immunology, Genetics and Pathology, Science for Life Laboratory, Rudbeck Laboratory, Uppsala University, Uppsala, Sweden (SA,NA,FP,AA); Department of Biotechnology, AlbaNova University Center, Royal Institute of Technology, Stockholm, Sweden (AK,SH)

**Keywords:** antibody, biotin, conjugation, protein detection, tissue microarray

## Abstract

Antibody-based protein profiling on a global scale using immunohistochemistry constitutes an emerging strategy for mapping of the human proteome, which is crucial for an increased understanding of biological processes in the cell. Immunohistochemistry is often performed indirectly using secondary antibodies for detection, with the benefit of signal amplification. Direct immunohistochemistry instead brings the advantage of multiplexing; however, it requires labeling of the primary antibody. Many antibody-labeling kits do not specifically target IgG and may therefore cause labeling of stabilizing proteins present in the antibody solution. A new conjugation method has been developed that utilizes a modified Z-domain of protein A (ZBPA) to specifically target the Fc part of antibodies. The aim of the present study was to compare the ZBPA conjugation method and a commercially available labeling kit, Lightning-Link, for in situ protein detection. Fourteen antibodies were biotinylated with each method and stained using immunohistochemistry. For all antibodies tested, ZBPA biotinylation resulted in distinct immunoreactivity without off-target staining, regardless of the presence of stabilizing proteins in the buffer, whereas the majority of the Lightning-Link biotinylated antibodies displayed a characteristic pattern of nonspecific staining. We conclude that biotinylated ZBPA domain provides a stringent method for antibody biotinylation, advantageous for in situ protein detection in tissues.

## Introduction

Mapping of the human proteome in normal and diseased cells will greatly increase our understanding of many aspects of cell biology, for example, differentiation and disease development, because proteins constitute the functional elements in most cell biological processes. Today, there are mainly two different strategies used for mapping of the human proteome: One is separation-based proteomics, which uses electrophoresis or liquid chromatography in combination with mass spectrometry to study proteins in complex bio samples, whereas the other is based on usage of affinity proteins to study proteins in various applications, for example, western blotting (WB) and immunohistochemistry (IHC). IHC allows for in situ visualization of protein expression in tissues and is a valuable tool in clinical pathology. The specificity and sensitivity with which an antibody binds its intended target is of fundamental importance to achieve reliable data.

A prerequisite for studying the proteome using the affinity-based proteomics approach is the availability of high-quality affinity proteins, most commonly antibodies. Unlike for DNA and RNA analysis, specific probes for the detection of particular proteins of interest cannot be generated with the same ease. The simplistic nature of complementary nucleic acid sequences hybridizing to specifically bind each other is far from the complexity of binding between antibodies and specific epitopes. A large-scale antibody-based proteomic project, The Human Protein Atlas program (www.proteinatlas.org), aims to generate affinity purified polyclonal antibodies toward all non-redundant human proteins (approx. 20,000 proteins) to map the human proteome using IHC in a wide range of normal and cancer tissues assembled in tissue microarrays (TMAs) (Uhlén et al. 2005; Pontén et al. 2011). The project thus offers a huge resource of validated antibodies, and, in the current version 11.0 of The Human Protein Atlas, antibodies toward proteins corresponding to 15,156 human genes have been used for protein expression profiling.

An antibody used in an assay is selected because of its ability to target a specific protein. However, all antibodies also display different degrees of affinity for additional proteins (off targets), and any staining pattern generated using IHC depends both on the kinetics of the antibody-target protein binding and on the relative amount of off target present in the analyzed specimen (Fritschy 2008; Bordeaux et al. 2010). This potentially causes cross-reactivity and complicates determination of the protein expression pattern. This is, of course, especially true for poorly characterized proteins for which there are no other data available. Adding to the complexity is the fact that validation of antibodies is both application- and context dependent, because the sample treatment associated with different methods causes target proteins and immunogenic epitopes to be in different states (Bordeaux et al. 2010). In addition, time before fixation, time of fixation, tissue processing, and the type of fixative used can all affect antigenicity and antibody performance (Fritschy 2008). IHC performed on a TMA does not eliminate all these issues but offers the possibility to minimize inter-experimental differences, as all samples are stained simultaneously under identical conditions (Kononen et al. 1998).

IHC is commonly performed using an indirect labeling technique, in which a secondary antibody is used carrying the reporter molecule or enzyme for visualization. A secondary antibody brings along the benefit of amplifying the signal from the bound primary antibody, making it very useful for bright field microscopy and routine pathology laboratory investigations. However, when looking into the kinetics of antibody–antigen binding, or when multiple antigens or multiple epitopes on the same antigen are to be targeted simultaneously, direct labeling of the primary antibodies is advantageous.

Biotin is a commonly used conjugate for antibodies. The subsequent detection is accomplished with avidin or, more frequently used today, streptavidin, coupled to a reporter molecule. The avidin-biotin complex technique was first exploited in the 1970s and has since been developed and become useful for in situ localization of antigens in cells and tissues (Becker and Wilchek 1972; Heitzmann and Richards 1974). The interaction shows low reversibility, with a dissociation constant (k_d_) of 4 × 10^−14^ M (Green 1975), thus permitting numerous combinations of avidin, biotin, and antibody. There are many commercial antibody-labeling kits on the market that are quick and convenient to use, but most often they require a fairly high antibody concentration. Moreover, the targets for labeling are often amine or carboxyl groups, rendering the labeling nonspecific, meaning that also the variable regions of an antibody may be conjugated and its binding properties altered. In addition, other proteins used as stabilizers in the antibody buffer will be conjugated as well, which might cause nonspecific staining in tissues.

A new antibody labeling technique, using the Z-domain from staphylococcal protein A, has been developed (Konrad et al. 2011), and it assures a stringent and specific labeling of the Fc part of antibodies (Deisenhofer 1981; Gouda et al. 1998; Konrad et al. 2011). The Z-domain has been synthesized with the amino acid analogue benzoylphenylalanine (BPA), which binds covalently to other amino acids upon UV exposure (Dorman and Prestwich 2000). Moreover, a reporting group, for example biotin, can be incorporated into the protein domain for detection. Using solid-phase peptide synthesis as a production strategy enables the incorporation of the biotin in a specific position within the protein. Therefore, labeled streptavidin can be used for detection of biotinylated antibodies. The high affinity and specificity of the streptavidin-biotin interaction permits diverse applications in immunology, histochemistry, affinity chromatography, and other areas.

In the present study, we have evaluated and compared two antibody biotinylation techniques, using 14 antibodies directed toward 13 different proteins, analyzed in 18 tissues. The Z-domain of protein A (ZBPA) biotinylation and the commercially available biotinylation kit, Lightning-Link, were compared using IHC based on streptavidin-horseradish peroxidase (HRP) detection on TMAs. We found that antibodies biotinylated with the ZBPA technique show a staining pattern in concordance with their unconjugated counterparts. ZBPA therefore offers a good approach to label antibodies for in situ protein detection.

## Materials & Methods

### Ethics Statement

The use of HPA tissue arrays in this study is covered by the HPA ethical permit (EPN Uppsala 2002/577, 2007/159). The investigators do not know the identities of arrayed tissue samples, nor will they be released into the public domain.

### Tissue Microarrays

A TMA block containing 25 cores representing 18 formalin fixed paraffin-embedded normal human tissues was used (Table 1). Tissues included in the TMA design were selected to be representative of the panel of proteins investigated. The diversity of tissues included in the TMA enabled evaluation of the IHC results in a wide range of different cell types, covering cell types expected to be positive as well as negative for each of the antibodies. The TMA block was produced as described earlier (Kampf et al. 2012), and 4-μm sections were cut and placed on Superfrost Plus microscope slides (Thermo Fisher Scientific; Fremont, CA) for IHC.

**Table 1.** Tissue Types and Number of Cores per Tissue for the Tissue Microarray.

Tissue Type	No. of Cores	Tissue Type	No. of Cores
Tonsil	2	Prostate	2
Uterus	2	Testis	1
Placenta	2	Cerebral cortex	1
Stomach	1	Cerebellum	1
Duodenum	1	Fallopian tube	2
Small intestine	1	Skin	1
Colon	1	Liver	2
Rectum	1	Kidney	1
Skeletal muscle	1	Pancreas	2

Tissues represented by two cores are from two different patients.

### Selection of Antibodies

In order to study the effect of biotinylation, well-validated antibodies toward proteins expressed in a variety of cell types and different subcellular localizations were selected from The Human Protein Atlas portal. Both affinity-purified polyclonal antibodies and mouse monoclonal antibodies were included, and in total, 14 antibodies were used for IHC staining, targeting 13 different proteins (Table 2). All antibodies have previously been validated with protein array, WB, and IHC and used for protein profiling in The Human Protein Atlas. The protein expression data, including underlying images, as well as antibody validation data are publicly available at www.proteinatlas.org. Biotin was conjugated to each antibody using two different protocols, the ZBPA-biotinylation technique and the commercially available biotin conjugation kit, Lightning-Link. All antibodies, with and without conjugated biotin, were titrated in order to yield a good signal-to-noise ratio within the set of 18 tissue types included in the tissue microarray. The summarized antibody-based protein expression pattern for each antibody is shown in Table 2.

**Table 2.** Antibodies Included in the Study.

		Antibody/Product ID			
Protein	Protein Function^a^	Species/Clonality	Distributor	Other Proteins Present	Primary Staining Location
ABCG1	transporter involved in macrophage lipid homeostasis	HPA031470 R PAb	Atlas Antibodies	albumin	cytoplasm in intestine, fallopian tube, uterus, testis, and skeletal muscle
ACAT1	plays a major role in ketone body metabolism	HPA007569 R PAb	Atlas Antibodies	albumin	mitochondria in most tissues
ACTL7B	ACTL7B is expressed predominantly in the testis; however, its exact function is not known.	HPA021803 R PAb	Atlas Antibodies	albumin	cytoplasm in testis
ANXA1	calcium/phospholipid-binding protein, which promotes membrane fusion and is involved in exocytosis	CAB035987 M MAb	NCI-CPTC	N/A	plasma membrane and cytoplasm in placenta, inflammatory cells, and prostate
		HPA011271 R PAb	Atlas Antibodies	albumin	plasma membrane and cytoplasm in placenta, inflammatory cells, and prostate
B2M	component of the class I major histocompatibility complex (MHC); involved in the presentation of peptide antigens to the immune system	CAB002572 R PAb	Novocastra	albumin	extracellular in prostate glandules
CALD1	actin- and myosin-binding protein implicated in the regulation of actomyosin interactions in smooth muscle and non-muscle cells	HPA017330 R PAb	Atlas Antibodies	albumin	cytoplasm in smooth muscle
CTCF	chromatin-binding factor that binds to DNA sequence-specific sites; involved in transcriptional regulation	AMAb90663 M MAb	Atlas Antibodies	N/A	nuclei in most tissues
KRT1	may regulate the activity of kinases such as protein kinase C (PKC) and SRC via binding to integrin beta-1 (ITB1) and the receptor of activated PKC (RACK1/GNB2L1)	HPA017917 R PAb	Atlas Antibodies	albumin	plasma membrane and cytoplasm in epidermis
MAP2	The exact function of MAP2 is unknown but MAPs may stabilize the microtubule (MT) against depolymerization.	HPA012828 R PAb	Atlas Antibodies	albumin	cytoplasm in neuronal cells and pancreatic islets
SIGLEC6	putative adhesion molecule that mediates sialic acid-dependent binding to cells	HPA018198 R PAb	Atlas Antibodies	albumin	plasma membrane in placenta
STMN1	involved in the regulation of the MT filament system by destabilizing MTs	CAB010107 M MAb	Santa Cruz Biotechnology	gelatin	cytoplasm in selected cells in tonsil, intestine, uterus, and testis
TOP2A	control of topological states of DNA by transient breakage and subsequent rejoining of DNA strands	HPA006458 R PAb	Atlas Antibodies	albumin	nuclei mainly in proliferating cells, e.g., in testis, tonsil, skin, and intestine
VIL1	epithelial cell-specific Ca(2+)-regulated actin-modifying protein that modulates the reorganization of microvillar actin filaments	HPA006884 R PAb	Atlas Antibodies	albumin	microvilli in intestinal epithelial cells and kidney proximal tubule cells

Antibody information, including antibody ID from The Human Protein Atlas or product ID for not yet published antibodies. M, mouse; R, rabbit.

a UniProt and NCBI databases; acquired June 19, 2013.

### Biotinylation with ZBPA

The reagent ZBPA biotin was produced by peptide synthesis as described in Konrad et al. (2011). The general procedure for photoconjugation was as follows. Solutions of 100 nM antibodies and 2 µM ZBPA biotin in PBS were incubated at 20C for 1 hr. Cross-linking was achieved by exposure to light, 365 nm (Spectronics Corporation; Westbury, NY), for 2 hr on ice. For buffer exchange and recovery of conjugated antibodies from excess ZBPA biotin, Vivaspin ultrafiltration spin columns with a 30 kDa cut-off (Vivaspin; Sartorius Stedim Biotech, Goettingen, Germany) were used, and centrifugation was performed at 15,000 rcf for 10 min. Buffers used were 0.2 M acetic acid (VWR; Radnor, PA), pH 3.2, for lowering the pH and PBST (0.01% Tween 20) for restoring the pH to 7. ZBPA-biotinylated antibodies were stored at 4C.

### Biotinylation with Lightning-Link

Antibodies were conjugated to biotin using the Lightning-Link Biotin Conjugation Kit Type A (Innova Biosciences; Cambridge, UK, ref. 704-0010) according to the procedure recommended by the manufacturer. In brief, 100 μl antibody was mixed with 10 μl LL-modifier reagent, which was then applied onto the lyophilized Lightning-Link mix and incubated for 3 hr at room temperature before adding 10 μl LL-quencher reagent. The protein concentration of the antibodies ranged from 0.03 mg/ml to 1 mg/ml. HSA (20 μg/ml in PBS) and gelatin (1 mg/ml in PBS) were also biotinylated with the same kit. LL-biotinylated antibodies, HSA, and gelatin were stored at 4C.

Six of the Lightning-Link-conjugated antibodies were also filtered using Vivaspin ultrafiltration spin columns with a 10 kDa cut-off (Sartorius Stedim Biotech) in order to dispose possible free biotin. The antibodies were diluted with PBS and centrifuged twice at 15,000 rcf for 10 min with the addition of PBS after each centrifugation.

### Automated Immunohistochemistry

TMA slides were deparaffinized in xylene, hydrated in graded alcohols, and blocked for endogenous peroxidase for 5 min in 0.3% H_2_O_2_ diluted in 95% ethanol. Heat-induced epitope retrieval (HIER) was done in a Decloacing chamber (Biocare Medical; Walnut Creek, CA) with citrate buffer, pH 6.0 (Thermo Fisher Scientific, ref. TA-250-PM1X), for 4 min at 125C. Before staining, the slides were immersed in wash buffer (Thermo Fisher Scientific, ref. TA-999-TT) containing 0.2% Tween 20 (Thermo Fisher Scientific, ref. TA-125-TW) for 15 min to avoid surface tension. Unconjugated antibodies were automatically stained in an Autostainer 480S instrument (Thermo Fisher Scientific) at room temperature with the following steps: UltraV block (Thermo Fisher Scientific, ref. TA-125-UB) for 5 min, primary antibody for 30 min, primary antibody enhancer (Thermo Fisher Scientific, ref. TL-125-PB) for 20 min (only for mouse antibodies), UltraVision LP HRP polymer (Thermo Fisher Scientific, ref. TL-125-PH) for 30 min, and diaminobenzidine (DAB; Thermo Fisher Scientific, ref. TA-125-HDX) for 5 min × 2. Between all incubations, the slides were rinsed in wash buffer (Thermo Fisher Scientific). The slides were counterstained with Mayer’s hematoxylin (Histolab; Gothenburg, Sweden, ref. 01820), dehydrated, and coverslipped using Pertex (Histolab, ref. 00871.0500).

### Manual Immunohistochemistry

Deparaffinization, peroxidase blocking, and HIER were performed as described above. The staining was performed manually at room temperature for all biotinylated antibodies to minimize the antibody volume used. The slides were blocked for endogenous biotin with Avidin/Biotin Blocking System (Thermo Fisher Scientific, ref. TA-015-BA) for 20 min each, followed by primary antibody for 30 min (to resemble the automated IHC protocol for unconjugated antibodies), Large Volume Streptavidin Peroxidase (Thermo Fisher Scientific, ref. TA-060-HR) for 10 min, and DAB for 10 min. Between all incubations, the slides were rinsed in wash buffer. The slides were counterstained and coverslipped as described above.

### Digital Imaging and Analysis

For each protein, unconjugated antibodies and ZBPA- and Lightning-Link-conjugated antibodies were titrated to yield a similar staining intensity to enable a comparison of immunostaining patterns. The immunostained TMA slides were digitized using Aperio ScanScope CS Slide Scanner system (Aperio Technologies; Vista, CA) at 40-fold magnification.

The IHC staining using unconjugated antibody was used as a reference when evaluating the two different biotin conjugation methods, to reveal any possible alterations in the staining pattern caused by the labeling of the antibody. Tissue and cellular distribution as well as subcellular localization of HRP positivity were recorded and used for a comparative analysis. To be regarded as similar, the overall positivity in the represented tissues, cell types, and subcellular structures needed to be unchanged regardless of biotinylation.

### Western Blot

Fifteen μg of RT-4 cell line lysate were subjected to a precast 4–20% Criterion SDS-PAGE gradient gel (Bio-Rad Laboratories; Hercules, CA) followed by transfer to a PVDF membrane using Criterion gel blotting sandwiches (Bio-Rad Laboratories) according to the manufacturer’s recommendations. PVDF membranes were presoaked in methanol and blocked (5% dry milk and 0.05% Tween 20 in TBS) for 45 min at room temperature followed by 1 hr of incubation with primary antibody diluted in blocking buffer. After three 5-min washes in TBST (0.05% Tween 20), the membranes were incubated for 1 hr with either an HRP-conjugated secondary antibody (polyclonal swine anti-rabbit antibody 1:3000, ref. P0399, or polyclonal goat anti-mouse antibody 1:7000, ref. P0447; Dako, Glostrup, Denmark) or Streptavidin Peroxidase (Thermo Fisher Scientific, ref. TS-060-HR). A final round of three 5-min TBST washes was performed before chemiluminescence detection, using a CCD camera (Bio-Rad Laboratories) and Immobilon Western Chemiluminescent HRP Substrate (Millipore Corporation; Billerica, MA).

## Results

A set of 14 antibodies was selected for displaying a reliable staining pattern using IHC in which the primary antibody was unconjugated, and a secondary antibody was used for detection. Two biotinylation assays, ZBPA and Lightning-Link, were used for direct labeling of the 14 different primary antibodies, and the resulting stainings in a diverse set of tissues were evaluated using the results from corresponding unconjugated antibodies.

### Unconjugated Antibodies

Unconjugated antibodies were used to stain TMAs to serve as references for the biotinylated antibodies. They were stained using a standardized IHC protocol with secondary antibodies coupled to an HRP polymer. The 14 different antibodies showed staining patterns in agreement with existing literature and/or with a paired antibody (data not shown). The staining localizations for all antibodies are summarized in Table 2 and are here described in brief. ACTL7B, ANXA1 (MAb and PAb), B2M, CALD1, KRT1, MAP2, SIGLEC6, and Villin1 displayed expression patterns characterized by distinct positivity in one or a few tissues, as seen for ACTL7B and KRT1, exclusively expressed in testis and skin, respectively. ABCG1, ACAT1, CTCF, STMN1, and TOP2A instead displayed a ubiquitous expression in several different tissues. Most proteins displayed expression in the nucleus, cytoplasm, and/or plasma membrane; however, ACAT1 is a mitochondrial protein expressed in most cell types, and Villin1 displayed expression exclusively in microvilli of cells in the intestinal epithelium and in kidney proximal tubules. The IHC staining patterns generated using these 14 antibodies were used as the references to evaluate the two conjugation methods.

### ZBPA Biotinylation

**Figure 1.**
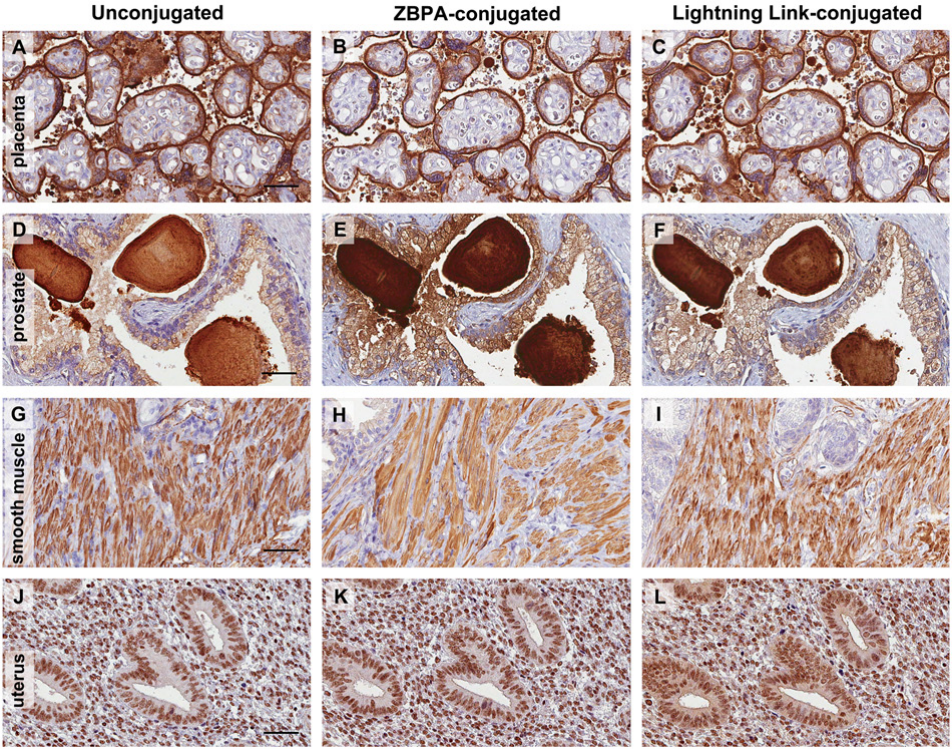
Z-domain of protein A (ZBPA)- and Lightning-Link-biotinylated antibodies displaying concordant immunohistochemical staining to the unconjugated equivalents. The antibodies target four different proteins, here exemplified in one representative tissue each: ANXA1 (MAb) in placenta (A–C), B2M in prostate (D–F), CALD1 in smooth muscle of prostate (G–I), and CTCF in uterus (J–L). Brown color indicates antibody binding, and hematoxylin (blue) was used as counterstaining. Bars = 50 µm.

**Figure 2.**
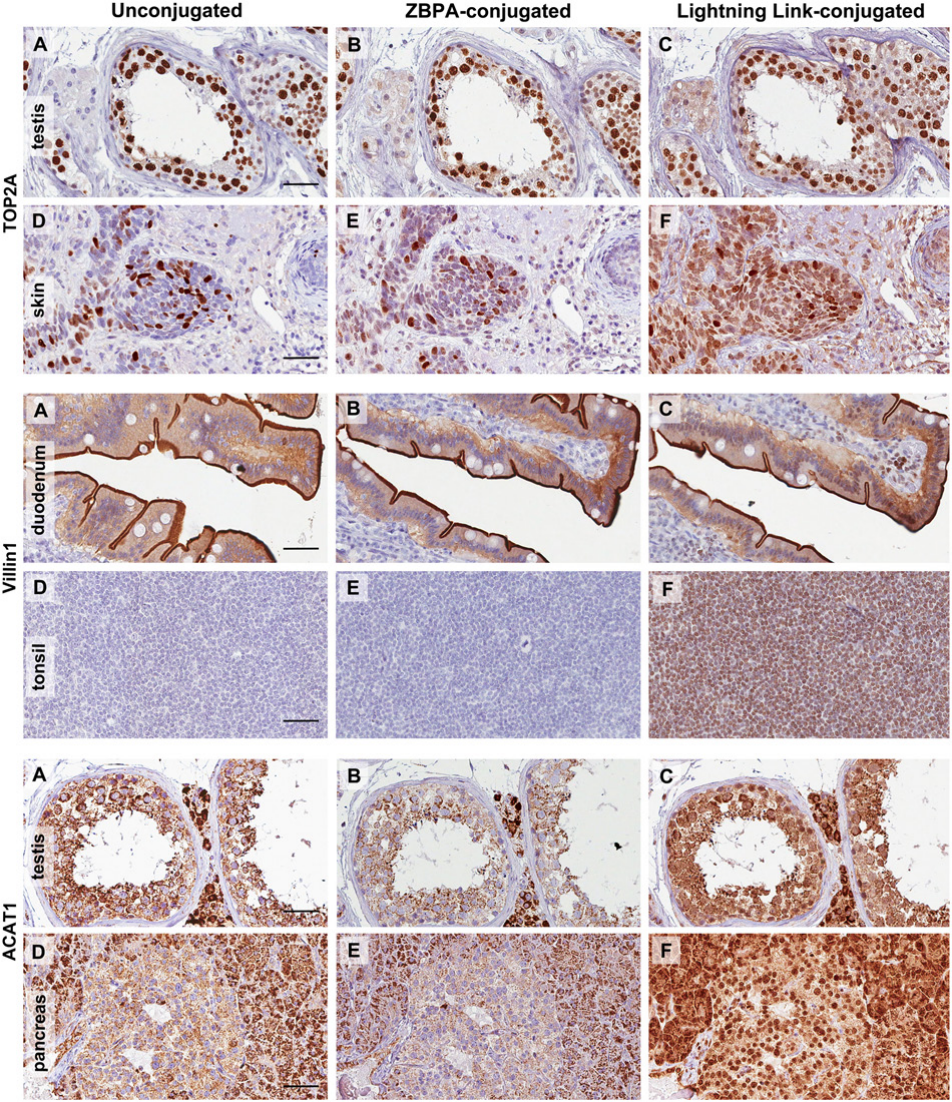
Immunohistochemical staining for antibodies toward five different proteins (TOP2A, Villin1, and ACAT1) is shown. For each antibody, one tissue is shown, in which both Z-domain of protein A (ZBPA)- and Lightning-Link (LL)-conjugated antibodies generate a staining in concordance with the unconjugated equivalent (A–C), and one tissue in which the LL-conjugated antibody results in additional off-target staining (D–F). Brown color indicates antibody binding, and hematoxylin (blue color) was used as counterstaining. Bars = 50 µm.

**Figure 3.**
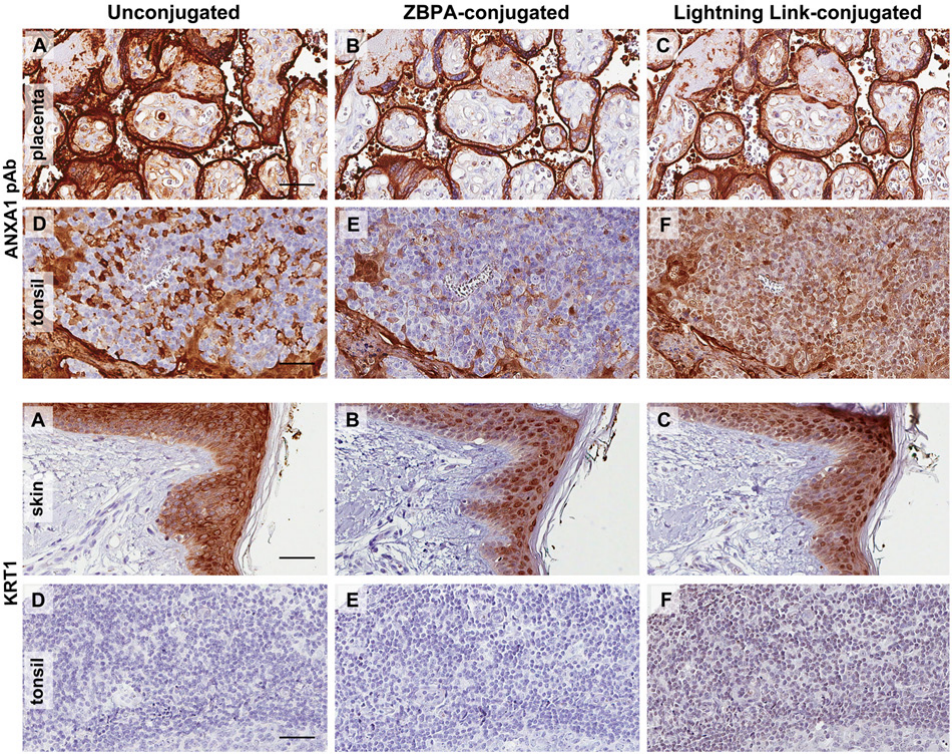
Immunohistochemical staining for antibodies toward five different proteins (ANXA1 PAb and KRT1) is shown. For each antibody, one tissue is shown, in which both Z-domain of protein A (ZBPA)- and Lightning-Link (LL)-conjugated antibodies generate a staining in concordance with the unconjugated equivalent (A–C), and one tissue in which the LL-conjugated antibody results in additional off-target staining (D–F). Brown color indicates antibody binding, and hematoxylin (blue color) was used as counterstaining. Bars = 50 µm.

**Figure 4.**
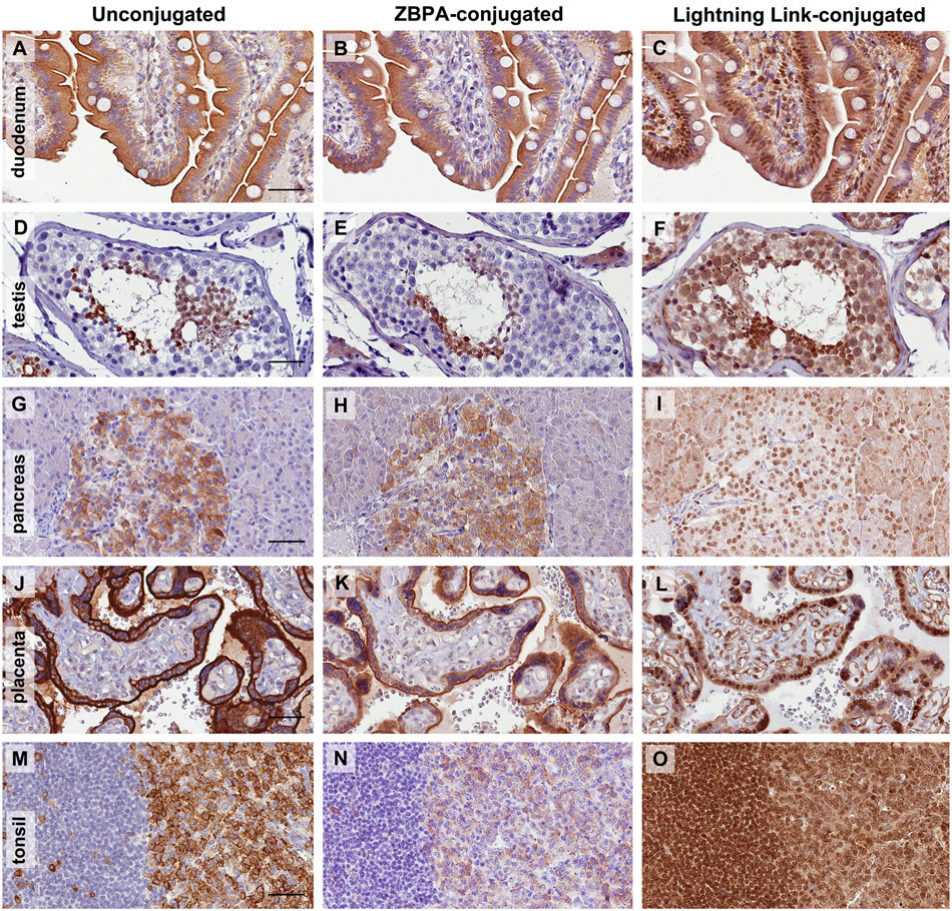
Off-target staining for five Lightning-Link (LL)-biotinylated antibodies and concordant staining for the corresponding Z-domain of protein A (ZBPA)-biotinylated antibodies compared with the unconjugated equivalents. The antibodies target ABCG1, shown in duodenum (A–C), ACTL7B in testis (D–F), MAP2 in pancreas (G–I), SIGLEC6 in placenta (J–L), and STMN1 in tonsil (M–O). The cytoplasmic staining seen in pancreatic islets with the unconjugated and ZBPA-biotinylated MAP2 antibody is absent for the LL-biotinylated antibody. Brown color indicates antibody binding, and hematoxylin (blue color) was used as counterstaining. Bars = 50 µm.

The staining patterns of ZBPA-biotinylated and corresponding unconjugated antibodies were compared. For all 14 antibodies, ZBPA biotinylation resulted in a staining pattern concordant with that of the unconjugated antibody. ANXA1 (MAb) displayed a cytoplasmic and membranous staining pattern most prominent in placenta, B2M showed secreted staining of concretions in prostate, CALD1 was seen in smooth muscle, and CTCF showed nuclear staining in several tissues (Fig. 1). TOP2A displayed nuclear staining in proliferating cells, Villin1 showed staining of villi in intestine and kidney, ACAT1 had a granular staining in most tissues, ANXA1 (PAb) showed a cytoplasmic and membranous staining pattern mainly in placenta (as did ANXA1 MAb), and KRT1 stained exclusively the epidermis of skin (each antibody is exemplified in two tissues as shown in Figs. 2 and 3). ABCG1 expression was seen in the cytoplasm of intestine; fallopian tube, and uterus, among others; ACTL7B was seen exclusively in seminiferous ducts of testicle; MAP2 showed cytoplasmic staining of neuronal cells and cells of pancreatic islets; SIGLEC6 showed cytoplasmic and membranous staining only in placenta; and STMN1 was positive in the cytoplasm of selected cells of tonsil, intestine, uterus, and testicle, although the staining intensity of ZBPA-biotinylated STMN1 antibody could not fully reach that of the unconjugated counterpart (each antibody is exemplified in one tissue as shown in Fig. 4).

### Lightning-Link Biotinylation

For the Lightning-Link-biotinylated antibodies, 4 out of 14 antibodies showed a staining pattern in concordance with the corresponding unconjugated antibody (ANXA1 MAb, B2M, CALD1, and CTCF; Fig. 1). Five out of 14 antibodies (TOP2A, Villin1, ACAT1, ANXA1 PAb, and KRT1) showed a similar immunostaining pattern to the unconjugated antibodies, but with additional staining to different degrees as exemplified in two different tissues for each antibody in Figs. 2 and 3. The additional staining, not seen for the unconjugated antibodies, displayed the common features of strong nuclear positivity in tonsil and cerebellum and nuclear and/or cytoplasmic positivity in uterus, placenta, intestine, cerebral cortex, and pancreas. The remaining 5 out of 14 antibodies yielded a different immunostaining pattern compared to their unconjugated counterparts (ABCG1, ACTL7B, MAP2, SIGLEC6, and STMN1; Fig. 4). Here, a different staining pattern is defined as a positivity not congruent with the staining pattern from the unconjugated antibody, due to either loss of staining or an extensive staining that makes the expected staining pattern less distinctive.

In order to exclude the possibility of free biotin molecules causing unwanted additional staining, a subset of six antibodies was filtered to dispose free biotin. They were selected to represent antibodies showing a dissimilar or partly similar staining pattern compared with the unconjugated equivalents. Filtered antibodies displayed similar staining patterns to the non-filtered counterparts (data not shown).

**Figure 5.**
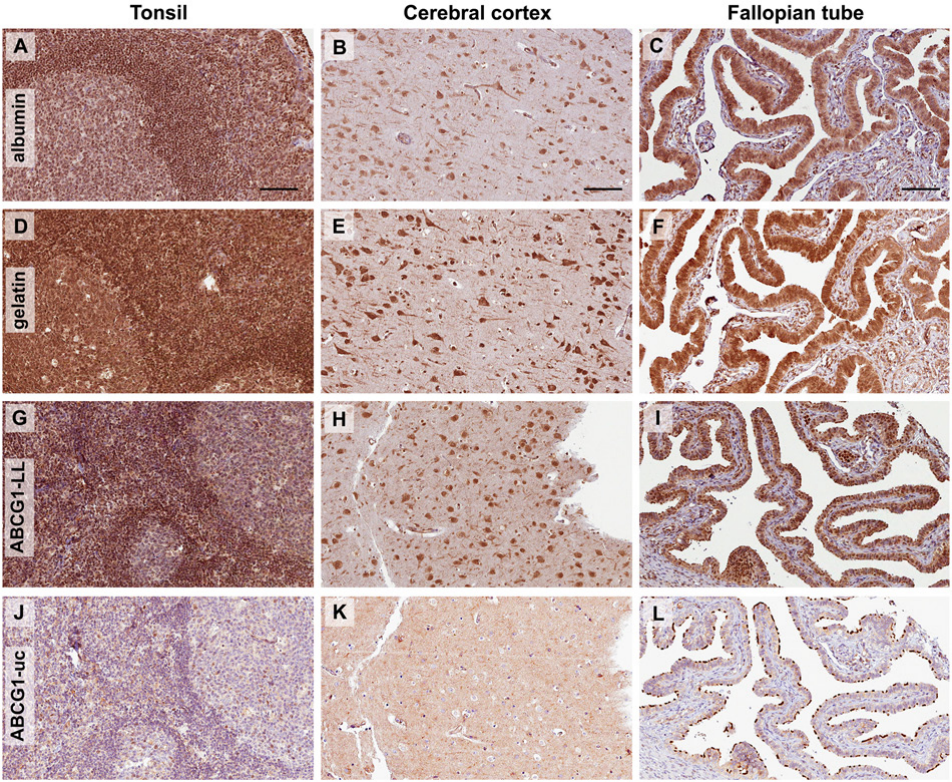
Histochemical staining with Lightning-Link (LL)-biotinylated human serum albumin and gelatin causing unspecific staining. The albumin (A–C) and gelatin (D–F) staining patterns resemble the off-target staining of many LL-biotinylated antibodies, here represented by an ABCG1 antibody (G–I). The results of the biotinylated proteins/antibody were compared with that of the unconjugated ABCG1 antibody (J–L) and shown in tonsil (A, D, G, and J), cerebral cortex (B, E, H, and K), and fallopian tube (C, F, I, and L). Brown color indicates HSA-, gelatin-, or antibody-binding, and hematoxylin (blue color) was used as counterstaining. Bars = 100 µm.

As a control for the effect of proteins often used as stabilizers in antibody buffers, HSA and gelatin were biotinylated with ZBPA and Lightning-Link, respectively, and used instead of primary antibody for manual IHC with streptavidin-HRP detection. Lightning-Link conjugation resulted in nonspecific nuclear and cytoplasmic staining in most tissues for both stabilizers, with a staining pattern similar to that seen for many Lightning-Link-biotinylated antibodies; for example, nuclear positivity in tonsil and cerebellum and nuclear and/or cytoplasmic positivity in uterus, placenta, intestine, cerebral cortex, and pancreas (exemplified in three different tissues as shown in Fig. 5). No staining was seen for ZBPA-conjugated HSA and gelatin (data not shown).

The estimated antibody concentration needed to generate the same staining intensity was generally higher for the ZBPA-biotinylated antibodies than for the Lightning-Link-biotinylated antibodies.

### Western Blot

Because the performance of antibodies often is application dependent, a subset of five ZBPA-biotinylated antibodies and corresponding unconjugated antibodies was also tested with WB to confirm that they work for other applications as well. For ACAT1, one band was detected at 45 kDa, for B2M at 14 kDa, and for KRT1 at 66 kDa. For ANXA1, two bands were detected, out of which the strongest was seen at 39 kDa (Supplemental Fig. S1). All antibodies, ZBPA-biotinylated and unconjugated, thus resulted in bands of the expected size, showing that ZBPA-biotinylation of antibodies doesn’t change their usability for WB.

## Discussion

Validation of antibodies is crucial when profiling in situ protein expression in tissues, and although WB as well as protein arrays offer information on antibody quality, validation of antibodies is ultimately application dependent. Validation of IHC therefore cannot be reliably confirmed through other means than by studying the correlation with RNA levels in corresponding cell type (Kiflemariam et al. 2012) and/or by parallel staining with two separate antibodies targeting non-overlapping epitopes of the same protein, denoted as paired antibodies, on consecutive TMA sections. The comparison between immunostaining patterns of paired antibodies provides an especially advantageous validation strategy for poorly characterized proteins (Paavilainen et al. 2008; Uhlen et al. 2010). Paired antibodies can further be applied simultaneously on one tissue slide using methods such as double IHC or proximity ligation assay (PLA) (Fredriksson et al. 2002). In this way, the proteomic landscape in situ could potentially be uncovered with high reliability and also interactions there within. However, depending on the species of antibodies used as well as other details in the setup of the assay, direct labeling of the antibodies may be necessary or desirable.

In the present study, we have evaluated two methods for the biotinylation of antibodies. The commercial Lightning-Link kit tested is convenient and, according to the manufacturer, it works well with most amine-free buffers. Moreover, the presence of azide (0.02–0.1%), BSA (0.1–1.0%) or gelatin (0.1%) should not affect the achieved result considerably. The conjugation efficiency is claimed to be very high, close to 100%, and purification of the conjugates should not be needed since a quencher deactivates the conjugating chemicals in the kit (www.innovabiosciences.com, acquired June 28, 2013). The other technique used was developed quite recently and is based on a synthesized Z-domain, which makes it possible to covalently couple to the Fc domain of the antibody by using the photoreactive molecule, BPA.

When comparing the staining patterns obtained using conventional IHC, that is, unconjugated primary antibody and secondary anti-rabbit or anti-mouse antibodies, with the results obtained with ZBPA-conjugated antibodies, the results were almost identical. In some instances, directly labeled antibodies generated a more limited and stringent staining of protein expression than conventional IHC (Figs. 1, 2, 3, and 4). Considering that the performance of antibodies is application dependent, we also tested a subset of ZBPA-conjugated antibodies (ACAT1, ANXA1 MAb and PAb, B2M, and KRT1) for WB. The results were identical to those generated with unconjugated antibodies, suggesting that the biotinylation did not alter their binding ability in this application (Supplemental Fig. S1).

The results obtained from using the Lightning-Link-conjugated antibodies, on the other hand, reveal that, for in situ detection using directly labeled IHC, this biotinylation method is suboptimal, because although stabilizing proteins like albumin and gelatin do not cause insufficient biotinylation of the antibodies, they instead cause nonspecific background staining. This is consistent with earlier observations stating that background staining is reduced if albumin is omitted (Mittelbronn et al. 2006). Eleven of the antibody samples examined in this study contained albumin of different concentrations, and one contained gelatin. Although no BSA is added to the polyclonal antibodies generated within the HPA project, it is known that a fraction of the rabbit serum albumin passes through the process of affinity purification, resulting in a mean albumin concentration of 13.5 μg/ml (data not shown). If labeled albumin hypothetically binds to proteins in the tissue section during immunostaining, it will inevitably result in unwanted staining.

IHC-staining results using the ZBPA-conjugated antibodies suggest that if the conjugation is specifically directed to the Fc part of antibodies, albumin or other unwanted proteins cannot be biotinylated, and more stringent immunostaining is possible. The lower staining intensity seen for ZBPA-biotinylated STMN1 (Fig. 4), compared with the unconjugated counterpart, is most likely due to a low antibody concentration caused by loss of antibody in the filtering step after conjugation. Furthermore, the IHC protocol used here is standardized for a high throughput proteomic profiling but could of course be further optimized regarding incubation times, antibody concentrations, and retrieval for individual antibodies. The staining intensity could possibly be increased by incorporating two biotin molecules in the Z-domain, because this potentially would double the detection efficiency; this, consequently, would also enable a lower amount of antibody needed for immunostaining. It is important to note is that the ZBPA technique also offers the possibility of conjugating other molecules than biotin to an antibody. In the situation of dual IHC using paired antibodies of the same species, the antibodies can be made distinguishable by the use of distinct conjugate molecules targeted by secondary antibodies.

Ten out of 14 Lightning-Link-conjugated antibodies yielded a common staining pattern superimposed to the expected protein expression profile for each antibody. This common staining pattern was characterized by, for example, nuclear positivity in tonsil and cerebellum and nuclear and/or cytoplasmic positivity in uterus, placenta, intestine, cerebral cortex, and pancreas. Most antibodies in this study were not within the concentration range recommended by the manufacturer for the Lightning-Link kit but were of lower concentrations, which may cause large excesses of free biotin molecules. However, in our hands, removal of free biotin through filtration did not alter the staining pattern compared with that observed using unfiltered antibodies (data not shown), suggesting that free unconjugated biotin is not the cause of the additional background staining. When albumin and gelatin were conjugated with Lightning-Link and used in the IHC setup, a pattern very similar to the additional background staining could be seen (Fig. 5).

Five of the Lightning-Link-conjugated antibodies showed an altered staining pattern overall (ABCG1, ACTL7B, MAP2, SIGLEC6, and STMN1; Fig. 4), which may be the effect of high albumin-to-antibody ratio, masking the expected staining. Also, it is possible that, for some antibodies, Lightning-Link affects the binding ability due to biotinylation in the Fab region. This might explain the loss of cytoplasmic staining in pancreatic islets for MAP2 after conjugation with Lightning-Link (Fig. 4).

Although the use of ZBPA-biotinylated antibodies does not prove superior to conventional IHC, stringent labeling increases the range of in situ detection methodologies for which antibodies can be used. For example, multiple antibodies raised in the same species may, with this technique, be distinguished from each other and thereby be used in various dual detection applications, such as proximity ligation assay. Apart from dual detection of one protein, PLA is also an excellent method for studying protein interactions and protein modification (Söderberg et al. 2006; Jarvius et al. 2007). Protein targets can be readily detected and localized with high specificity and sensitivity with single molecule resolution and objectively quantified in unmodified cells and tissues. This would greatly increase the reliability protein localization, which is of enormous value for basal cell biology and studies of disease processes on a cellular level.

In conclusion, stringent conjugation is of great importance, as it widens the repertoire of techniques for which antibodies can be used. The ZBPA biotinylation is a highly specific conjugation method that does not cause any nonspecific staining for IHC, independently of stabilizing proteins in the buffer. Lightning-Link is a convenient labeling method that requires no additional purification steps. However, for antibodies used for in situ protein detection in tissues, it requires an antibody buffer free from other proteins to avoid nonspecific staining. ZBPA is therefore the preferred labeling technique for in situ protein detection in tissues.

## References

[bibr1-0022155413502360] Becker JM Wilchek M 1972 Inactivation by avidin of biotin-modified bacteriophage. Biochim Biophys Acta. 264(1):165–170 4553809 10.1016/0304-4165(72)90127-4

[bibr2-0022155413502360] Bordeaux J Welsh A Agarwal S Killiam E Baquero M Hanna J Anagnostou V Rimm D 2010 Antibody validation. BioTechniques. 48(3):197–209. 10.2144/000113382 20359301 PMC3891910

[bibr3-0022155413502360] Deisenhofer J 1981 Crystallographic refinement and atomic models of a human Fc fragment and its complex with fragment B of protein A from Staphylococcus aureus at 2.9- and 2.8-A resolution. Biochemistry. 20(9):2361–2370 7236608

[bibr4-0022155413502360] Dorman G Prestwich GD 2000 Using photolabile ligands in drug discovery and development. Trends Biotechnol. 18(2):64–77 10652511 10.1016/s0167-7799(99)01402-x

[bibr5-0022155413502360] Fredriksson S Gullberg M Jarvius J Olsson C Pietras K Gústafsdóttir SM Ostman A Landegren U 2002 Protein detection using proximity-dependent DNA ligation assays. Nat Biotechnol. 20(5):473–477. 10.1038/nbt0502-473 11981560

[bibr6-0022155413502360] Fritschy JM 2008 Is my antibody-staining specific? How to deal with pitfalls of immunohistochemistry. Eur J Neurosci. 28(12):2365–2370. 10.1111/j.1460-9568.2008.06552.x 19087167

[bibr7-0022155413502360] Gouda H Shiraishi M Takahashi H Kato K Torigoe H Arata Y Shimada I 1998 NMR study of the interaction between the B domain of staphylococcal protein A and the Fc portion of immunoglobulin G. Biochemistry. 37(1):129–136. 10.1021/bi970923f 9425032

[bibr8-0022155413502360] Green NM 1975 Avidin. Adv Protein Chem. 29:85–133 237414 10.1016/s0065-3233(08)60411-8

[bibr9-0022155413502360] Heitzmann H Richards FM 1974 Use of the avidin-biotin complex for specific staining of biological membranes in electron microscopy. Proc Natl Acad Sci U S A. 71(9):3537–3541 4139715 10.1073/pnas.71.9.3537 PMC433809

[bibr10-0022155413502360] Jarvius M Paulsson J Weibrecht I Leuchowius KJ Andersson AC Wählby C Gullberg M Botling J Sjöblom T Markova B 2007 In situ detection of phosphorylated platelet-derived growth factor receptor beta using a generalized proximity ligation method. Mol Cell Proteomics. 6(9):1500–1509. 10.1074/mcp.M700166-MCP200 17565975

[bibr11-0022155413502360] Kampf C Olsson I Ryberg U Sjöstedt E Pontén F 2012 Production of tissue microarrays, immunohistochemistry staining and digitalization within the human protein atlas. J Vis Exp. (63). 10.3791/3620 PMC3468196 22688270

[bibr12-0022155413502360] Kiflemariam S Andersson S Asplund A Pontén F Sjöblom T 2012 Scalable in situ hybridization on tissue arrays for validation of novel cancer and tissue-specific biomarkers. PloS One. 7(3):e32927. 10.1371/journal.pone.0032927 22412953 PMC3297615

[bibr13-0022155413502360] Kononen J Bubendorf L Kallioniemi A Bärlund M Schraml P Leighton S Torhorst J Mihatsch MJ Sauter G Kallioniemi OP 1998 Tissue microarrays for high-throughput molecular profiling of tumor specimens. Nat Med. 4(7):844–847 9662379 10.1038/nm0798-844

[bibr14-0022155413502360] Konrad A Karlström AE Hober S 2011 Covalent immunoglobulin labeling through a photoactivable synthetic Z domain. Bioconjug Chem. 22(12):2395–2403. 10.1021/bc200052h 22026370

[bibr15-0022155413502360] Mittelbronn M Dietz K Simon P Beschorner R Schleich A Nguyen TD Meyermann R Schlaszus H 2006 Albumin in immunohistochemistry: foe and friend. Appl Immunohistochem Mol Morphol. 14(4):441–444. 10.1097/01.pai.0000203040.79156.70 17122643

[bibr16-0022155413502360] Paavilainen L Wernérus H Nilsson P Uhlén M Hober S Wester K Pontén F 2008 Evaluation of monospecific antibodies: a comparison study with commercial analogs using immunohistochemistry on tissue microarrays. Appl Immunohistochem Mol Morphol. 16(5):493–502. 10.1097/PAI.0b013e31817c645e 18685494

[bibr17-0022155413502360] Pontén F Schwenk JM Asplund A Edqvist PH 2011 The Human Protein Atlas as a proteomic resource for biomarker discovery. J Intern Med. 270(5):428–446. 10.1111/j.1365-2796.2011.02427.x 21752111

[bibr18-0022155413502360] Söderberg O Gullberg M Jarvius M Ridderstråle K Leuchowius KJ Jarvius J Wester K Hydbring P Bahram F Larsson LG 2006 Direct observation of individual endogenous protein complexes in situ by proximity ligation. Nat Methods. 3(12):995–1000. 10.1038/nmeth947 17072308

[bibr19-0022155413502360] Uhlén M Björling E Agaton C Szigyarto CA Amini B Andersen E Andersson AC Angelidou P Asplund A Asplund C 2005 A Human Protein Atlas for normal and cancer tissues based on antibody proteomics. Mol Cell Proteomics. 4(12):1920–1932. 10.1074/mcp.M500279-MCP200 16127175

[bibr20-0022155413502360] Uhlen M Oksvold P Fagerberg L Lundberg E Jonasson K Forsberg M Zwahlen M Kampf C Wester K Hober S 2010 Towards a knowledge-based Human Protein Atlas. Nat Biotechnol. 28(12):1248–1250. 10.1038/nbt1210-1248 21139605

